# Outcomes and Regional Care Patterns of Critically Ill Pediatric Emergency Patients: A Population‐Based Registry Study

**DOI:** 10.1111/ped.70545

**Published:** 2026-09-08

**Authors:** Noriyuki Kaku, Kanako Higashi, Go Kawano, Masashi Kobayashi, Jun Muneuchi, Shinichi Morimoto, Kenji Furuno, Junpei Mukai, Jun Kono, Wakato Matsuoka, Soichi Mizuguchi, Kenichiro Yamamura, Yasunari Sakai, Tomohiko Akahoshi

**Affiliations:** ^1^ Department of Pediatrics, Graduate School of Medical Sciences Kyushu University Fukuoka Japan; ^2^ Department of Advanced Emergency and Disaster Medicine, Graduate School of Medical Sciences Kyushu University Fukuoka Japan; ^3^ Emergency and Critical Care Center Kyushu University Hospital Fukuoka Japan; ^4^ Department of Pediatrics St. Mary's Hospital Kurume Japan; ^5^ Department of Pediatrics Kitakyushu City Yahata Hospital Kitakyushu Japan; ^6^ Department of Pediatrics Kyushu Hospital, Japan Community Healthcare Organization Kitakyushu Japan; ^7^ Department of Emergency and Critical Care Medicine, Faculty of Medicine Fukuoka University Fukuoka Japan; ^8^ Department of General Pediatrics & Interdisciplinary Medicine Fukuoka Children's Hospital Fukuoka Japan; ^9^ Department of Pediatrics and Child Health Kurume University School of Medicine Kurume Japan

**Keywords:** child death review, critically ill children, epidemiology, hospital case volume, pediatric emergency care

## Abstract

**Background:**

Clarifying clinical features of critically ill pediatric patients is essential for optimizing the regional emergency care system. However, the relationships between hospital case volume, interhospital transfer, and outcomes, including causes of death, remain unclear.

**Methods:**

An observational study was conducted using prospectively collected data on critically ill patients aged 15 years or younger. Patients enrolled in this study were those who met the criteria for emergency and critical care unit management in Fukuoka Prefecture, Japan, from June 2014 to May 2019. Data on etiologic categories, outcomes, interhospital transfer, and use of intensive care interventions were collected. Hospitals were classified according to patient volume (high‐volume: ≥ 100 and low‐volume: < 100 patients during the observation period).

**Results:**

Among 3124 patients, 891 (28.5%) were infants (< 1 year). The annual incidence was 0.85 per 1000 children. Endogenous, exogenous, and “undetermined” etiologies accounted for 2481 (79.4%), 495 (15.8%), and 148 (4.7%) patients, respectively. Of 327 deaths (10.5%), 157 (48.0%) occurred in infants, and 89 (56.7%) of infant deaths were classified as “undetermined”. In‐hospital mortality among admitted patients was higher in low‐volume hospitals (8.7% vs. 5.3%, *p* < 0.01), as was the proportion of “undetermined” deaths (49.5% vs. 28.8%, *p* < 0.01). Transferred patients had higher ICU admission rates (62.5% vs. 52.6%, *p* < 0.01), without higher mortality among admitted patients.

**Conclusions:**

Critically ill infants had high mortality, and many deaths remained undetermined. These findings highlight the need to strengthen regional transport systems and postmortem evaluation in pediatric emergency care.

**Abbreviations**
CPAOA: cardiopulmonary arrest on arrival
ECMO: extracorporeal membrane oxygenation
ER: emergency room
ICU: intensive care unit
PICU: pediatric intensive care unit
RRT: renal replacement therapy

## 1 Introduction

Critically ill pediatric patients require emergency service for respiratory failure, shock, or cardiac arrest [1]. These patients represent a relatively small proportion of pediatric emergency cases but are associated with a substantial risk of mortality and severe morbidity, highlighting the need for well‐organized emergency and intensive care services [2, 3]. Thus, timely access to appropriate critical care and accurate identification of underlying causes are assumed to be essential for improving outcomes of critically ill pediatric patients.

A population‐level understanding of the incidence, clinical outcomes, and underlying etiologies of critical illness is expected to inform the organization of pediatric emergency and critical care [4, 5, 6]. Therefore, in Japan, epidemiological studies have examined critically ill pediatric emergency patients through cross‐institutional surveys conducted within defined regions, providing valuable information on incidence, mortality, and factors associated with adverse outcomes [7, 8]. However, it remains unclear how hospital case volume and interhospital transfer within regional care systems relate to outcomes and the distribution of etiologic categories, especially among fatal cases.

In this study, we conducted a population‐based registry analysis using prospectively collected data on critically ill pediatric emergency patients across the largest geographic area reported to date. Using a prefecture‐wide registry that involved all pediatric departments in Fukuoka Prefecture, we sought to provide a more complete picture of disease burden, outcomes, and etiologic categories, with particular emphasis on fatal cases, and examine the association between hospital case volume and mortality.

## 2 Methods

### 2.1 Study Design and Data Collection

We conducted a registry‐based observational study using prospectively collected data on critically ill pediatric emergency patients aged 15 years or younger who were transported to hospitals in Fukuoka Prefecture on Kyushu Island, in the southern part of Japan. Kyushu Island covers an area of 36,782 km^2^ with a population of 13,016,329, which accounted for approximately 10% of the total Japanese population as of 2015 [9]. Fukuoka Prefecture had a population of 5,101,556, with 724,329 (14.2%) aged 15 years or younger in 2015 (Figure S1). All hospitals with pediatric departments in Fukuoka Prefecture (96 facilities) participated in the study (see Appendix A). All 96 hospitals cooperated in the registry and were eligible for case registration. During the study period, 24 of these hospitals had at least one child who met the predefined criteria for critical illness and was attributed to that hospital in the final analytic dataset; therefore, these 24 hospitals constituted the denominator for analyses of hospital case volume. The mechanically ventilated subgroup analysis was restricted to 19 hospitals that had at least one mechanically ventilated patient in the final analytic dataset. A standardized survey form was completed monthly for pediatric emergency patients judged to be seriously ill at each hospital and submitted to the Fukuoka Prefectural Office. The survey form included information on age group (< 6 months, 7 months to 1 year, 1–4 years, 5–9 years, and 10–15 years), gender, disease category (endogenous, exogenous, or undetermined; endogenous disorders were further classified as neurological, respiratory, cardiovascular, gastrointestinal, renal, hematological, endocrine/metabolic, congenital, or other endogenous disorders), cardiopulmonary arrest on arrival (CPAOA), involvement of abuse or infection, intensive care unit (ICU; as defined by the Japanese government's insurance policy) admission, therapeutic device usage (mechanical ventilation, renal replacement therapy [RRT], extracorporeal membrane oxygenation [ECMO]), and outcome (alive or deceased). Interhospital transfer was defined as transfer from another hospital prior to admission to the participating facility. The survey was conducted by the Committee for Pediatric Emergency Medicine of Fukuoka Prefecture. The collected data were analyzed at Kyushu University Hospital.

### 2.2 Study Population

Critically ill pediatric emergency patients aged 15 years or younger who were transported to hospitals in Fukuoka Prefecture between June 1, 2014, and May 31, 2019, were enrolled. The study period preceded the COVID‐19 pandemic and was therefore not affected by its social and healthcare impacts. Critically ill patients were defined as patients who met the criteria for application of the “Emergency and Critical Care Unit Management Fee” under the Japanese national health insurance system, including patients who died in the emergency room (ER). These criteria include impaired consciousness or coma, acute respiratory failure, acute heart failure, acute drug intoxication, shock, severe metabolic disorders (e.g., liver failure, renal failure, or severe diabetes), extensive burns, status after emergency major surgery, post‐resuscitation status, and other serious conditions such as trauma and tetanus. Patients admitted to the neonatal intensive care unit (NICU), patients undergoing scheduled surgery, and end‐stage patients with malignant diseases were not registered. In Japan, dedicated pediatric intensive care units (PICUs) are limited in number, and critically ill children are frequently managed in general ICUs that provide care for both adult and pediatric patients, or occasionally outside designated ICUs, depending on resource availability and institutional practices.

**FIGURE 1:**
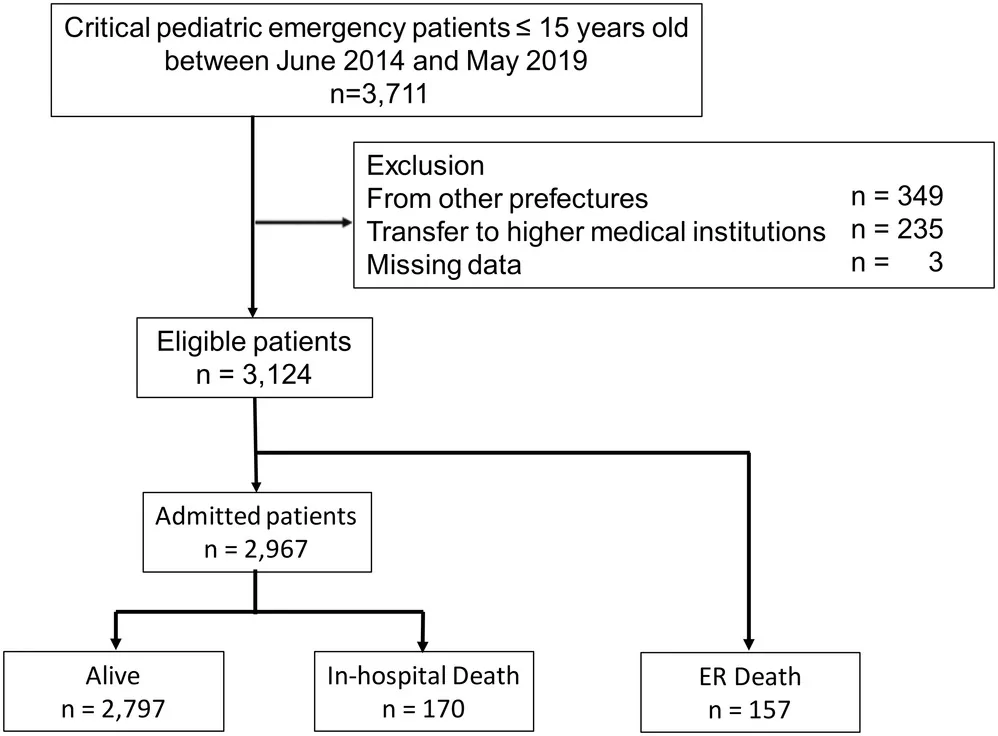
Flowchart of patient enrollment. Clinical information was collected from 3711 critically ill pediatric emergency patients aged ≤ 15 years transported to participating hospitals between June 2014 and May 2019. Patients from other prefectures, those transferred to higher‐level hospitals to avoid duplicate registration, and those with missing data were excluded. The remaining 3124 eligible patients were included in the analysis and classified according to clinical outcomes and hospital case volume. The annual incidence of critically ill pediatric emergency cases was 0.85 per 1000 children; this was calculated using 3124 cases over 5 years and the 2015 population of children aged ≤ 15 years in Fukuoka Prefecture.

We excluded patients from other prefectures because they were outside the predefined study population. In addition, patients transferred to higher‐level hospitals were excluded to avoid duplicate registration, as these patients were registered at the referred tertiary hospitals. Transfers occurred either directly from the emergency department or after hospital admission, depending on the clinical course (Figure 1).

Hospitals were categorized as high‐ or low‐volume using an operational cutoff of 100 critically ill pediatric cases over the five‐year study period, selected for pragmatic stratification in the absence of established volume thresholds in pediatric emergency care. Informed consent was obtained using an opt‐out approach. This study was approved by the Institutional Review Board of Kyushu University Hospital (#26‐73).

### 2.3 Statistical Analyses

Differences in the distribution of categorical variables were analyzed using the chi‐squared test or Fisher's exact test, as appropriate. Continuous variables were assessed using the Mann–Whitney *U*‐test. *p* values < 0.05 were considered to indicate statistical significance. Univariable and multivariable logistic regression analyses were performed to identify factors associated with mortality among admitted patients. Variables with a *p* value < 0.10 in univariable analyses, as well as clinically relevant variables, were considered for inclusion in the multivariable models. Two multivariable models were constructed: Model 1 included baseline demographic and clinical variables, and Model 2 additionally included organ support variables. Odds ratios (ORs) with 95% confidence intervals (CIs) were calculated. All statistical analyses were performed using the JMP Pro software program (ver. 14.2.0; SAS Institute, Cary, NC, USA).

## 3 Results

### 3.1 Demographics and Clinical Profile of Patients

During the five‐year study period, 3711 pediatric emergency patients were initially enrolled. After excluding 349 patients from other prefectures, 235 patients transferred to higher‐level hospitals, and three patients with missing data, 3124 patients were analyzed and categorized into three groups: survivors, deceased in the hospital, and deceased in the ER (Figure 1). The annual incidence of critically ill pediatric emergency cases was 0.85 per 1000 children; this was calculated using 3124 cases over 5 years and the 2015 population of children aged ≤ 15 years in Fukuoka Prefecture. The demographic and clinical characteristics of the study population are shown in Table 1. Overall, 327 patients (10.5%) died during the study period. Among the patients, 891 (28.5%) were younger than 1 year, and this age group accounted for nearly half of all deaths (157/327, 48.0%). Endogenous disorders accounted for 79.4% of all cases, whereas exogenous categories accounted for 15.8%, and 4.7% were classified as undetermined. Neurological and respiratory disorders were the most common endogenous conditions (Table S1). Cardiopulmonary arrest on arrival (CPAOA) and the presence of infection also varied significantly between the groups. Among hospitalized patients, 54.7% were admitted to the ICU (as defined by insurance policy). ICU admission, mechanical ventilation, RRT, and ECMO were significantly more frequent among patients who died.

**TABLE 1:** Demographics of the critically ill pediatric emergency patients.

	All *N* = 3124	Survivor *N* = 2797	In‐hospital death *N* = 170	ER death *N* = 157	*p*
Age groups, *n*					**< 0.01**
0‐6 month	571 (18.3)	460 (16.4)	41 (24.1)	70 (44.6)	
7 month‐1 year	320 (10.2)	274 (9.8)	22 (12.9)	24 (15.3)	
1–4 years	1133 (36.3)	1042 (37.3)	52 (30.6)	39 (24.8)	
5–9 years	587 (18.8)	556 (19.9)	21 (12.4)	10 (6.4)	
10–15 years0‐	513 (16.4)	465 (16.6)	34 (20.0)	14 (8.9)	
Infant (< 1 year), *n*	891 (28.5)	733 (26.2)	63 (37.1)	94 (59.9)	**< 0.01**
Male, *n*	1707 (54.6)	1530 (54.7)	98 (57.6)	79 (50.3)	0.41
Etiologic category, *n*					**< 0.01**
Endogenous	2481 (79.4)	2334 (83.4)	111 (65.3)	36 (22.9)	
Exogenous	495 (15.8)	430 (15.4)	34 (20.0)	31 (19.7)	
Undetermined	148 (4.7)	33 (1.2)	25 (14.7)	90 (57.3)	
CPAOA, *n*	256 (8.2)	59 (2.1)	61 (35.9)	136 (86.6)	**< 0.01**
Abuse, *n*	53 (1.7)	45 (1.6)	3 (1.8)	5 (3.2)	0.33
Infection, *n*	1162 (37.2)	1092 (39.0)	52 (30.6)	18 (11.5)	**< 0.01**
Interhospital transfer, n	637 (20.4)	595 (21.3)	39 (22.9)	3 (1.9)	**< 0.01**
ICU admission^a^, *n*	1623 (54.7)	1487 (53.2)	136 (80.0)	NA	**< 0.01**
Mechanical ventilation^a^, *n*	1195 (40.3)	1041 (37.2)	154 (90.6)	NA	**< 0.01**
Renal replacement therapy^a^, *n*	118 (4.0)	93 (3.3)	25 (14.7)	NA	**< 0.01**
ECMO^a^, *n*	40 (1.3)	30 (1.1)	10 (5.9)	NA	**< 0.01**

*Note:* Values in parentheses indicate percentage. Interhospital transfer in Table 1 was calculated among all registered critically ill children, including patients who died in the ER. The bold indicate statistically significant *p* values (*p* < 0.05).

Abbreviations: CPAOA, Cardiopulmonary arrest on arrival; ECMO, Extracorporeal membrane oxygenation; ICU, Intensive care unit; NA, Not available.

^a^ ICU admission, mechanical ventilation, renal replacement therapy, and ECMO are presented as numbers and percentages among hospitalized patients.

### 3.2 Hospital Case Volume and Patient Distribution

**FIGURE 2:**
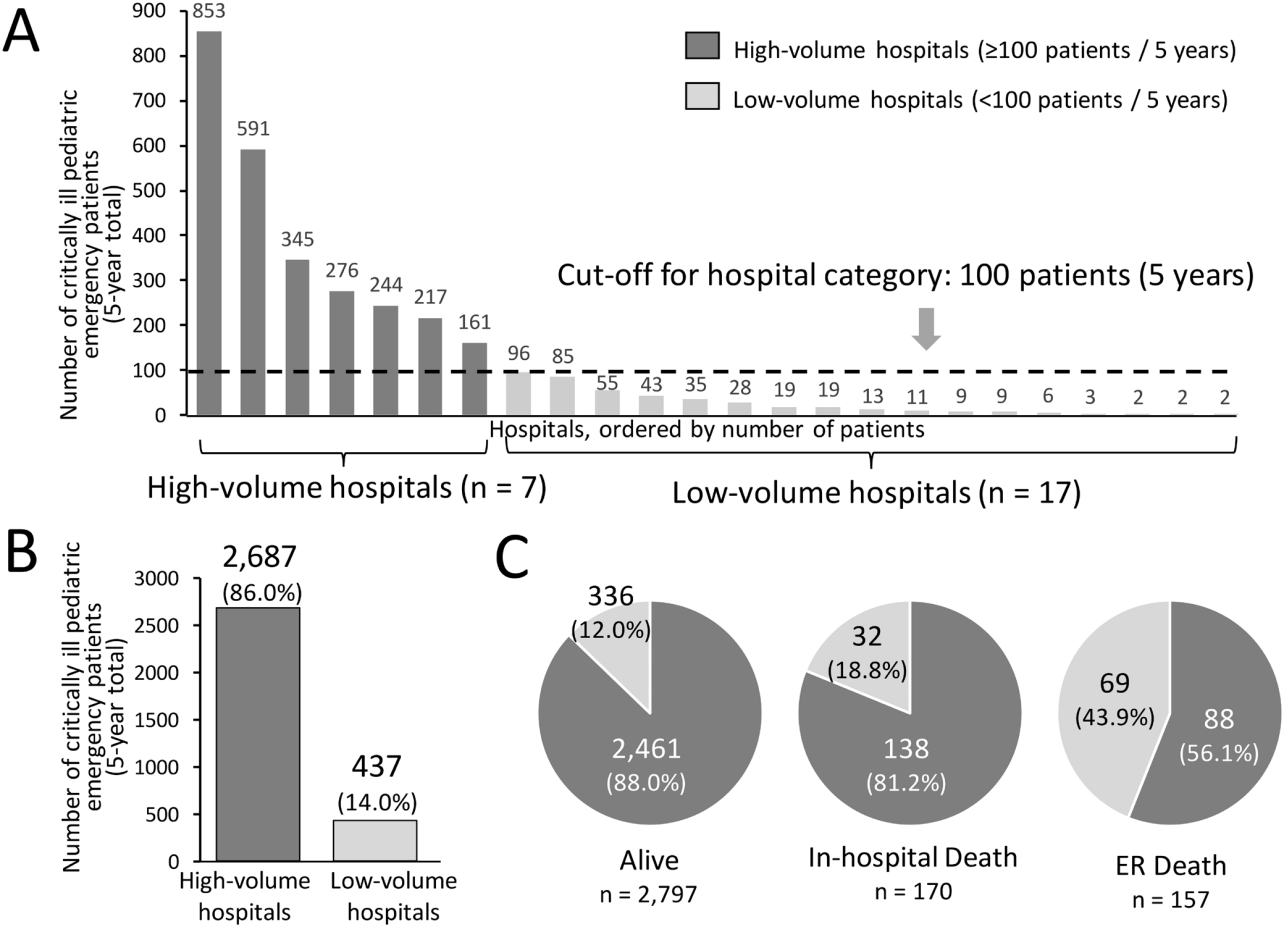
Distribution of critically ill pediatric emergency patients and outcomes according to hospital case volume. (A) Number of patients per hospital (*n* = 24), ordered by case volume. The dashed line indicates the cut‐off of 100 patients over 5 years. Figure 2A includes the 24 hospitals that had at least one critically ill child attributed to them in the final analytic dataset. Hospitals among the 96 participating facilities without any attributed critically ill patients were not included in the hospital‐volume analysis. (B) Proportion of total patients in high‐volume hospitals (≥ 100 patients over 5 years; 86.0%) and low‐volume hospitals (< 100 patients; 14.0%). (C) Distribution of outcomes (alive, in‐hospital death, and ER death) by hospital case volume (dark gray, high‐volume hospitals; light gray, low‐volume hospitals).

**FIGURE 3:**
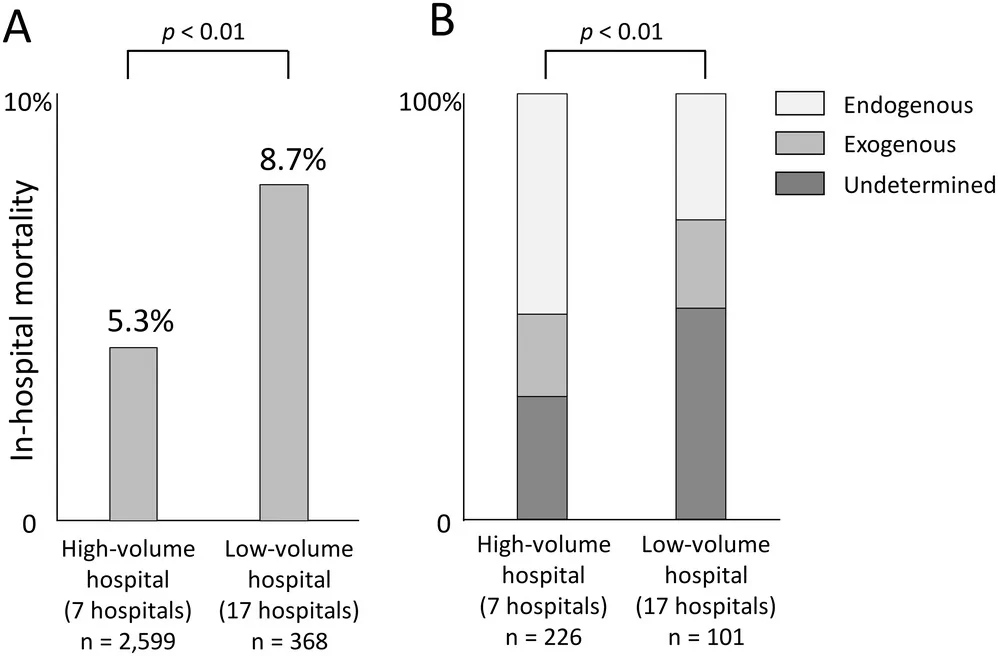
Mortality and etiologic categories according to hospital case volume. Comparison of mortality and etiologic categories of death between high‐volume hospitals (≥ 100 critically ill pediatric patients over 5 years) and low‐volume hospitals (< 100 patients). (A) In‐hospital mortality rates. (B) Distribution of etiologic categories among all deceased patients.

The distribution of patient volume across hospitals was highly skewed, with a limited number of hospitals treating the majority of critically ill pediatric emergency patients (Figure 2). Hospitals were categorized according to case volume into high‐volume (≥ 100 patients over the 5‐year study period) and low‐volume (< 100 patients) groups. High‐volume hospitals comprised 7 of 24 hospitals, whereas the remaining 17 hospitals were classified as low‐volume. Most patients (86.0%) were treated in high‐volume hospitals; however, deaths—particularly those occurring in the emergency department—were disproportionately observed in low‐volume hospitals. Patients treated at low‐volume hospitals had significantly higher in‐hospital mortality than those treated at high‐volume hospitals (8.7% vs. 5.3%, *p* < 0.01). In addition, deaths in low‐volume hospitals were more frequently classified as undetermined than those in high‐volume hospitals (49.5% vs. 28.8%, *p* < 0.01) (Figure 3A,B). Among mechanically ventilated patients, high‐volume hospitals had higher ICU admission rates and greater use of advanced therapies such as RRT and ECMO. In contrast, mortality was not significantly different between hospital groups despite these differences in resource utilization (Table S2).

### 3.3 Interhospital Transfer and Resource Utilization

Among patients admitted to the hospital, 634 of 2967 (21.4%) were interhospital transfers (Table 2). Compared with direct admission, transferred patients more frequently required intensive care, including ICU admission (62.5% vs. 52.6%) and mechanical ventilation (54.3% vs. 36.4%) (all *p* < 0.01). Despite the higher need for intensive care interventions, mortality among admitted patients did not differ significantly between the transfer and direct admission groups (6.2% vs. 5.6%) (Figure S2).

**TABLE 2:** Demographics of the critically ill pediatric emergency patients according to transfer status.

	All *N* = 2967	Interhospital transfer *N* = 634	Direct admission *N* = 2333	*p*
Age groups, *n*				**< 0.01**
0‐6 month	501 (16.9)	137 (21.6)	364 (15.6)	
7 month‐1 year	296 (10.0)	80 (12.6)	216 (9.3)	
1–4 years	1094 (36.9)	219 (34.5)	875 (37.5)	
5–9 years	577 (19.4)	107 (16.9)	470 (20.1)	
10–15 years	499 (16.8)	91 (14.4)	408 (17.5)	
Infant (< 1 year), *n*	796 (26.8)	217 (34.2)	579 (24.8)	**< 0.01**
Male, *n*	1626 (54.8)	346 (54.6)	1280 (54.9)	0.95
Etiologic category, *n*				**< 0.01**
Endogenous	2445 (82.4)	546 (86.1)	1899 (81.4)	
Exogenous	464 (15.6)	79 (12.5)	385 (16.5)	
Undetermined	58 (2.0)	9 (1.4)	49 (2.1)	
CPAOA, *n*	120 (4.0)	30 (4.7)	90 (3.9)	**< 0.01**
Abuse, *n*	48 (1.6)	11 (1.7)	37 (1.6)	0.95
Infection, *n*	1144 (38.6)	225 (35.5)	919 (39.4)	0.27
ICU admission, *n*	1623 (54.7)	396 (62.5)	1227 (52.6)	**< 0.01**
Mechanical ventilation, *n*	1195 (40.3)	344 (54.3)	851 (36.5)	**< 0.01**
Renal replacement therapy, *n*	118 (4.0)	35 (5.5)	83 (3.6)	**0.03**
ECMO, *n*	40 (1.3)	18 (2.8)	22 (0.9)	**< 0.01**

*Note:* Values in parentheses indicate percentage. Table 2 was restricted to patients admitted to the hospital and therefore excluded ER deaths. Accordingly, interhospital transfer was calculated among admitted patients. The bold indicate statistically significant *p* values (*p* < 0.05).

Abbreviations: CPAOA, Cardiopulmonary arrest on arrival; ECMO, Extracorporeal membrane oxygenation; ICU, Intensive care unit.

### 3.4 Mortality and Etiologic Categories of Death

**FIGURE 4:**
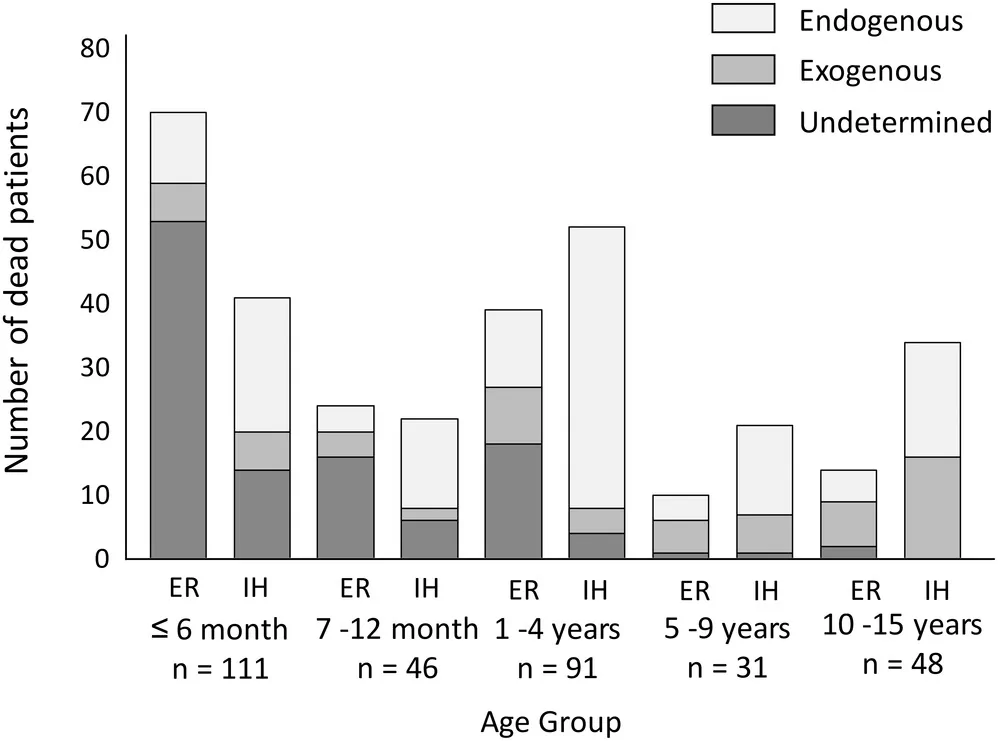
Etiologic categories of death by age group and location. Etiologic categories of death among pediatric emergency patients, stratified by age group and location of death. Deaths were classified as occurring in the emergency room or in‐hospital, and etiologies were categorized as endogenous, exogenous, or undetermined. ER, ER death; IH, In‐hospital death.

The demographic and clinical characteristics of the 327 deceased patients are summarized in Table 3. Overall, more than one‐third of all deaths were classified as having an undetermined etiology (115/327, 35.2%). Among all deceased patients, 157 (48.0%) died in the ER. The same number of deceased patients were infants (157/327, 48.0%). Deaths from undetermined causes accounted for 57.3% of ER deaths (Tables 1 and 3). Among infant deaths, 89 cases (56.7%) were classified as having an undetermined etiologic category. Across all age groups, deaths occurring in the ER were more frequently classified as undetermined than those occurring after hospital admission (Figure 4).

**TABLE 3:** Demographics of the deceased patients.

	All *N* = 327	High‐volume hospitals *N* = 226	Low‐volume hospitals *N* = 101	*p*
Hospitals, *n*	24	7	17	
Number of patients per hospital, *n*, median, range	11.5, 1–76	32.5, 10–76	4, 1–28	
Age groups, *n*				0.91
6 month	111 (33.9)	75 (33.2)	36 (35.6)	
7 month‐1 year	46 (14.1)	30 (13.3)	16 (15.8)	
1–4 years	91 (27.8)	66 (29.2)	25 (24.8)	
5–9 years	31 (9.5)	22 (9.7)	9 (8.9)	
10–15 years	48 (14.7)	33 (14.6)	15 (14.9)	
Infant (< 1 year), *n*	157 (48.0)	105 (46.5)	52 (51.5)	0.40
Male, *n*	176 (53.8)	122 (54.0)	54 (53.5)	0.93
Etiologic category, *n*				**< 0.01**
Endogenous	147 (45.0)	117 (51.8)	30 (29.7)	
Exogenous	65 (19.9)	44 (19.5)	21 (20.8)	
Undetermined	115 (35.2)	65 (28.8)	50 (49.5)	
CPAOA, *n*	197 (60.2)	124 (54.9)	73 (72.3)	**< 0.01**
Abuse, *n*	8 (2.4)	3 (1.3)	5 (5.0)	0.05
Infection, *n*	70 (21.4)	46 (20.4)	24 (23.8)	0.49
Interhospital transfer, *n*	42 (12.8)	37 (16.3)	5 (5.0)	**< 0.01**
Hospital admission, *n*	170 (52.0)	138 (61.1)	32 (31.7)	**< 0.01**
ICU admission^a^, *n*	136 (80.0)	107 (77.5)	29 (90.6)	0.10
Mechanical ventilation^a^, *n*	154 (90.6)	128 (92.8)	26 (81.3)	**0.04**
Renal replacement therapy^a^, *n*	25 (14.7)	24 (17.4)	1 (3.1)	**0.04**
ECMO^a^, *n*	10 (5.9)	10 (7.2)	0 (0.0)	0.12

*Note:* Values in parentheses indicate percentage. The hospital‐volume analysis included 24 hospitals that had at least one critically ill child attributed to them in the final analytic dataset among the 96 participating hospitals with pediatric departments in Fukuoka Prefecture. The bold indicate statistically significant *p* values (*p* < 0.05).

Abbreviations: CPAOA, Cardiopulmonary arrest on arrival; ECMO, Extracorporeal membrane oxygenation; ICU, Intensive care unit.

^a^ ICU admission, mechanical ventilation, renal replacement therapy, and ECMO are presented as numbers and percentages among hospitalized patients.

### 3.5 Factors Associated With Mortality

In univariable analyses, CPAOA, infant age (< 1 year), infection, admission to a low‐volume hospital, and organ support therapies were associated with mortality, whereas interhospital transfer was not (Table S3). The results of the multivariable analyses are shown in Table S4. Because the multivariable models were restricted to admitted patients, ER deaths were not included in these analyses. After adjustment for baseline factors (Model 1), CPAOA and admission to a low‐volume hospital were independently associated with mortality. After additional adjustment for organ support variables (Model 2), mechanical ventilation, RRT, and admission to a low‐volume hospital remained independently associated with mortality, while the association with CPAOA was attenuated but remained significant. Infant age (< 1 year) was not retained as an independent predictor in the multivariable models.

## 4 Discussion

In this regional population‐based study, mortality among critically ill pediatric emergency patients was high, particularly in infants, and many were managed outside intensive care units. A substantial proportion of deaths were undetermined, and in‐hospital mortality among admitted patients was associated with hospital case volume. Transferred patients required more intensive care but did not have higher mortality, suggesting that outcomes reflect both clinical severity and system‐level factors.

The overall mortality rate in this study was 10.5%, which is comparable to that reported in a previous Japanese study [8]. Notably, infants younger than 1 year accounted for approximately one‐third of all patients but nearly half of all deaths, highlighting the particular vulnerability of this age group among critically ill pediatric emergency patients. These findings suggest that early recognition and timely management of critical illness in infants may be particularly important to improve outcomes.

In countries with established pediatric intensive care systems, critically ill children are commonly managed in centralized PICUs [10, 11]. Reported PICU admission rates, including planned postoperative admissions, range from approximately 1.4–1.7 per 1000 children per year, although rates vary by country and region. Non‐elective admissions account for approximately two‐thirds of these admissions [4, 5, 6]. In contrast, recent Japanese studies have reported the incidence of critically ill pediatric emergency patients to be 0.64–0.84 per 1000 children per year [7, 8]. In the present study, which included more than ten times as many cases as prior domestic reports, the incidence was 0.85 per 1000 children per year, consistent with existing Japanese data.

Using national population statistics from 2015 (approximately 17.08 million children aged 15 years or younger), the annual number of critically ill pediatric emergency patients in Japan is estimated to be approximately 14,500. However, national surveys of PICU utilization indicate that only about 4900 critically ill pediatric emergency patients are admitted to PICUs annually [12], suggesting that current PICU bed capacity is insufficient to accommodate all such patients.

Consistent with this observation, only approximately 55% of critically ill pediatric emergency patients in the present study were admitted to an ICU, and even among those requiring mechanical ventilation, the ICU admission rate was approximately 75%. Dedicated PICU care was limited, with pediatric intensive care largely provided in general ICUs caring for both adult and pediatric patients. This pattern suggests substantial constraints in both ICU availability and pediatric critical care specialization in Japan [13]. Together, these findings highlight the need to define PICU admission criteria, support safe pediatric care in general ICUs, and expand pediatric critical care capacity where possible [2, 14].

In analyses of factors associated with mortality, cardiopulmonary arrest on arrival (CPAOA) was strongly associated with death, underscoring the critical importance of initial disease severity. After adjustment for organ support therapies, admission to low‐volume hospitals remained independently associated with mortality. However, mechanical ventilation and RRT may reflect both illness severity and subsequent treatment intensity and may also lie on the causal pathway to death; therefore, the adjusted estimate for low hospital volume in Model 2 should be interpreted cautiously and may not represent the total association between hospital volume and mortality. Moreover, because the multivariable analyses were restricted to admitted patients, they did not capture ER deaths, which accounted for a substantial proportion of deaths in low‐volume hospitals. Taken together, these findings suggest that the observed volume–mortality association should not be considered a direct measure of institutional quality.

In this study, low‐volume hospitals appeared to have lower ICU admission rates among mechanically ventilated patients and a higher proportion of deaths occurring in the emergency department. Although most patients were treated in high‐volume hospitals, deaths—particularly those in the emergency department—were disproportionately observed in low‐volume hospitals. This pattern likely reflects regional transport practices, in which patients with immediately life‐threatening conditions, such as CPAOA or persistent seizures, are appropriately transported first to the nearest hospital rather than distant tertiary centers. In such cases, safe interhospital transfer may not be possible, and the higher mortality observed in low‐volume hospitals may partly reflect the unavoidable concentration of patients with conditions too severe for transfer rather than differences in institutional quality alone.

Despite requiring more intensive care interventions, transferred patients did not have higher in‐hospital mortality than those admitted directly. These findings suggest that the association between hospital case volume and mortality [15] is influenced not only by institutional experience and resources but also by regional triage and transport systems [16, 17]. Improving outcomes will require timely triage, coordinated interhospital transfer [18], and transport systems supported by experienced personnel and specialized teams [19, 20, 21]. Importantly, these results do not diminish the essential role of low‐volume hospitals, which frequently provide the first life‐saving interventions for critically ill children.

Finally, more than one‐third of all deaths, over half of infant deaths, and the majority of ER deaths had undetermined etiologic categories. This high proportion likely reflects both limitations in diagnostic evaluation at or after death and the rare and heterogeneous nature of life‐threatening pediatric conditions, even among previously healthy children [22]. Previous studies have demonstrated that re‐evaluation of clinical information, genetic testing, and metabolic investigations can elucidate causes previously considered unexplained [23, 24]. The epidemiologic data presented here highlight the need to strengthen comprehensive postmortem evaluation and to develop Child Death Review (CDR) systems [25] that can objectively review the cause‐of‐death investigation process, identify gaps in postmortem assessment, and inform prevention strategies and pediatric emergency care in Japan.

## 5 Limitation

This study has several limitations. First, although this registry covered all pediatric departments in Fukuoka Prefecture, some eligible cases may have been missed because case registration depended on administrative billing criteria and monthly reporting by each participating site. Second, underlying diseases and long‐term outcomes, including neurological status after discharge, were not collected and could not be evaluated. Third, the study population was defined according to administrative eligibility criteria for emergency and critical care unit management under the Japanese health insurance system, which are not based on physiological severity scores; therefore, objective severity indices such as the Pediatric Index of Mortality score were outside the scope of data collection, and residual confounding by illness severity cannot be excluded. Fourth, hospital case volume was defined using an operational cutoff that was not based on outcome modeling, and alternative thresholds may yield different quantitative results. However, the overall pattern of patient concentration in high‐volume hospitals and higher mortality observed in low‐volume hospitals was consistent within the study cohort. Finally, detailed data on transport pathways and timing were not collected. Despite these limitations, this large, population‐based regional registry study provides important data to inform pediatric emergency and critical care in Japan.

## 6 Conclusion

This study revealed that nearly half of all deaths occurred in infants, and approximately half of critically ill children were managed outside intensive care units. In addition, many deaths remained of undetermined cause. Low hospital volume was associated with higher in‐hospital mortality among admitted patients and requires cautious interpretation. These findings support strengthening regional transport systems and postmortem evaluation to improve pediatric emergency care.

## Author Contributions

N.K. conceptualized and designed the study, drafted the initial manuscript, designed the data collection instruments, collected the data, and conducted the initial analyses. K.H., J.K., W.M., and S.Miz. collected the data and conducted the initial analyses. G.K., M.K., J.Mun., S.Mor., K.F., and J.Muk. collected the data and critically reviewed and revised the manuscript. K.Y., Y.S., and T.A. supervised the study and critically reviewed and revised the manuscript. All authors approved the final manuscript as submitted and agreed to be accountable for all aspects of the work.

## Funding

The work was supported in part by Japan Society for the Promotion of Science (JSPS) KAKENHI Grant numbers JP24K13391 (Kaku), JP25K14454 (Matsuoka) and JP23K15618 (Mizuguchi).

## Ethics Statement

This study was conducted in compliance with the institutional guidelines for clinical research. The specific protocol for the registry‐based observational analysis was approved by the Institutional Review Board of Kyushu University Hospital (#26–73). Informed consent was obtained in the form of an opt‐out option on a website.

## Conflicts of Interest

The authors declare no conflicts of interest.

## Supporting information


**Table S1:** Etiologic categories of the patients.
**Table S2:** Demographics of the mechanically ventilated patients.
**Table S3:** Univariable analysis of factors associated with mortality in hospitalized patients.
**Table S4:** Multivariable analysis of factors associated with mortality in hospitalized patients.

**Figure S1:** Study area and population. Geographic location and population characteristics of the study area, including Japan, Kyushu Island, and Fukuoka Prefecture. Black circles indicate high‐volume hospitals, and white circles indicate low‐volume hospitals.

**Figure S2:** Care pathways of critically ill pediatric emergency patients according to interhospital transfer status. Percentages were calculated within each admission pathway. ICU admission and mechanical ventilation are presented as proportions within each group. Mortality among admitted patients did not differ between groups despite higher use of intensive care interventions in transferred patients.

## Data Availability

The data that support the findings of this study are available on request from the corresponding author. The data are not publicly available due to privacy or ethical restrictions.

## Appendix A

Cooperating Hospitals are Abe Hospital, Arimatsu Hospital, Ariyoshi Hospital, Asakura Medical Association Hospital, Ashiya Central Hospital, Aso Iizuka Hospital, Chidoribashi Hospital, Chikugo City Hospital, Fukuoka Central Hospital, Fukuoka Children's Hospital, Fukuoka Dental College Medical and Dental Hospital, Fukuoka Kinen Hospital, Fukuoka Sanno Hospital, Fukuoka Shin Mizumaki Hospital, Fukuoka Tokushukai Hospital, Fukuoka Torikai Hospital, Fukuoka University Chikushi Hospital, Fukuoka University Hospital, Fukuokaken Saiseikai Futsukaichi Hospital, Fukuyoshi Hospital, Futsukaichi Nakagawa Hospital, Hakuaikai Kaita Hospital, Hamanomachi Hospital, Himeno Hospital, Hojo Ryoikuen Ikigai, Hospital of the University of Occupational and Environmental Health, Iizuka City Hospital, Japan Community Healthcare Organization Kurume General Hospital, Japan Community Healthcare Organization Kyushu Hospital, Japan Self Defense Forces Fukuoka Hospital, Japanese Red Cross Fukuoka Hospital, Jikeisone Hospital, Kama Red Cross Hospital, Kaneko Hospital, Kaneyuki Hospital, Kasuya Shinkouen Hospital, Kawasaki Municipal Hospital, KenAi Memorial Hospital, Kenwakaiotemachi Hospital, Keyaki‐kai Higashi Hospital, Kitakyushu City Yahata Hospital, Kitakyushu General Hospital, Kitakyushu Municipal Medical Center, Kitakyushu Rehabilitation Center for Children with Disabilities, Komenoyama Hospital, Konishidaiichi Hospital, Kurate Hospital, Kurume University Hospital, Kurume University Medical Center, Misaki Hospital, Mito Hospital, Miyata Hospital, Mizumaki Kyoritsu Hospital, Moji Hospital, Munakata Medical Association Hospital, Munakata Suikokai General Hospital, Murakami Hospital, Muta Hospital, National Hospital Organization Fukuoka National Hospital, National Hospital Organization Fukuokahigashi Medical Center, National Hospital Organization Kokura Medical Center, National Hospital Organization Kyushu Cancer Center, National Hospital Organization Kyushu Medical Center, National Hospital Organization Omuta National Hospital, Nishino Hospital, Okabe Hospital, Omuta Central Hospital, Omuta City Hospital, Omuta Tenryo Hospital, Onga Nakama Medical Association Onga Hospital, Public Yame General Hospital, Saiseikai Fukuoka General Hospital, Saiseikai Yahata General Hospital, Social Medical Corporation the Chiyukai Foundation Fukuoka Wajiro Hospital, Soujinkai Chikugoyoshii Cocoro Hospital, St. Joseph Welfare Center, St.Mary's Hospital, Steel Memorial Yawata Hospital, Sugahara Hospital, Suihoku Daiichi Hospital, Tachiarai Hospital, Tagawa Hospital, Tagawa Municipal Hospital, Takagi Hospital, Terasawa Hospital, Tobata General Hospital, Tohwa Hospital, Wakamatsu Hospital of the University of Occupational and Environmental Health, Yamabiko Gakuen, Yanagawa Hospital, Yanagawa Institute for Developmental Disabilities, Yanagawa Rehabilitation Hospital, and Yukari Rehabilitation Center.
