# Supplementary material for: Outcomes and Regional Care Patterns of Critically Ill Pediatric Emergency Patients: A Population‐Based Registry Study
Source: Pediatr Int. 2026 Sep 8;68(1):e70545. doi: 10.1111/ped.70545 (PMC13551226; doi:10.1111/ped.70545)
Supplement: Supplementary file 1 [file PED-68-e70545-s001.docx]

**Supplementary Table 1.** Etiologic categories of the patients

|  | All N = 3,124 | Alive N = 2,797 | In-hospital death N = 170 | ER death N = 157 | *p* value |
| --- | --- | --- | --- | --- | --- |
| **Endogenous, n** | 2,481 (79.4) | 2,334 (83.4) | 111 (65.3) | 36 (22.9) | **<0.01** |
| Neurological disorders | 1,045 (33.5) | 1,024 (36.6) | 19 (11.2) | 2 (1.3) |  |
| Respiratory disorders | 846 (27.1) | 808 (28.9) | 30 (17.6) | 8 (5.1) |  |
| Cardiovascular disorders | 212 (6.8) | 180 (6.4) | 23 (13.5) | 9 (5.7) |  |
| Gastrointestinal disorders | 132 (4.2) | 119 (4.3) | 11 (6.5) | 2 (1.3) |  |
| Renal disorders | 65 (2.1) | 62 (2.2) | 3 (1.8) | 0 (0.0) |  |
| Hematological disorders | 48 (1.5) | 43 (1.5) | 5 (2.9) | 0 (0.0) |  |
| Endocrine / Metabolic disorders | 55 (1.8) | 49 (1.8) | 5 (2.9) | 1 (0.6) |  |
| Congenital disorders | 45 (1.4) | 24 (0.9) | 13 (7.6) | 8 (5.1) |  |
| Other endogenous disorders | 33 (1.1) | 25 (0.9) | 2 (1.2) | 6 (3.8) |  |
| **Exogenous, n** | 495 (15.8) | 430 (15.4) | 34 (20.0) | 31 (19.7) |  |
| **Undetermined, n** | 148 (4.7) | 33 (1.2) | 25 (14.7) | 90 (57.3) |  |

Values in parentheses indicate percentage.

**Supplementary Table 2.** Demographics of the mechanically ventilated patients

|  | All N = 1,195 | High-volume hospitals N = 999 | Low-volume hospitals N = 196 | *p* value |
| --- | --- | --- | --- | --- |
| Hospitals, n | 19 | 7 | 12 |  |
| Number of patients per hospital, n, median, range | 38, 1-348 | 105, 75-348 | 11.5, 1-55 |  |
| Age groups, n |  |  |  | **<0.01** |
| - 6 month | 251 (21.0) | 225 (22.5) | 26 (13.3) |  |
| 7 month - 1 year | 132 (11.0) | 123 (12.3) | 9 (4.6) |  |
| 1-4 year | 425 (35.6) | 340 (34.0) | 85 (43.4) |  |
| 5-9 year | 201 (16.8) | 150 (15.0) | 51 (26.0) |  |
| 10-15 year | 186 (15.6) | 161 (16.1) | 25 (12.8) |  |
| Infant (<1 year), n | 383 (32.1) | 348 (34.8) | 35 (17.9) | **<0.01** |
| Male, n | 645 (54.0) | 542 (54.3) | 103 (52.6) | 0.66 |
| Etiologic category, n |  |  |  | 0.32 |
| Endogenous | 1,019 (85.3) | 857 (85.8) | 162 (82.7) |  |
| Exogenous | 134 (11.2) | 106 (10.6) | 28 (14.3) |  |
| Undetermined | 42 (3.5) | 36 (3.6) | 6 (3.1) |  |
| CPAOA, n | 101 (8.5) | 87 (8.7) | 14 (7.1) | 0.47 |
| Abuse, n | 21 (1.8) | 18 (1.8) | 3 (1.5) | 0.79 |
| Infection, n | 386 (32.3) | 327 (32.7) | 56 (28.6) | 0.47 |
| Interhospital transfer, n | 344 (28.8) | 322 (32.2) | 22 (11.2) | **<0.01** |
| ICU admission, n | 903 (75.6) | 788 (78.9) | 115 (58.7) | **<0.01** |
| Renal replacement therapy, n | 95 (7.9) | 92 (9.2) | 3 (1.5) | **<0.01** |
| ECMO, n | 38 (3.2) | 37 (3.7) | 1 (0.5) | **0.02** |
| Outcome |  |  |  |  |
| Death, n | 154 (12.9) | 128 (12.8) | 26 (13.3) | 0.86 |

Values in parentheses indicate percentage.

CPAOA, Cardiopulmonary arrest on arrival. ICU, Intensive care unit. ECMO, Extracorporeal membrane oxygenation.

This analysis was restricted to 19 hospitals that had at least one mechanically ventilated patient in the final analytic dataset among the 24 hospitals included in the hospital-volume analysis.

**Supplementary Table 3.** Univariable analysis of factors associated with mortality in hospitalized patients

| Variables | Total, n | Death, n | OR　（95% CI） | *p* value |
| --- | --- | --- | --- | --- |
| Male | 1,626 | 97 (6.0) | 1.1 (0.81 - 1.51) | 0.54 |
| Infant (< 1 year) | 796 | 63 (7.9) | 1.66 (1.20 - 2.29) | **<0.01** |
| CPAOA | 120 | 61 (50.8) | 26 (17.3 - 39.0) | **<0.01** |
| Abuse | 48 | 3 (6.3) | 0.91 (0.28 - 2.96) | 0.88 |
| Infection | 1,144 | 52 (4.6) | 1.45 (1.03 - 2.03) | **0.03** |
| Interhospital transfer | 634 | 39 (6.2) | 1.01 (0.69 - 1.46) | 0.97 |
| Low-volume hospital | 368 | 32 (8.7) | 1.7 (1.14 - 2.53) | **0.01** |
| ICU admission | 1,623 | 136 (8.4) | 3.52 (2.40 - 5.17) | **<0.01** |
| Mechanical ventilation | 1,195 | 154 (12.9) | 16.2 (9.64 - 27.3) | **<0.01** |
| Renal replacement therapy | 118 | 25 (21.2) | 5.01 (3.13 - 8.04) | **<0.01** |
| ECMO | 40 | 10 (25.0) | 5.77 (2.77 - 12.0) | **<0.01** |

ORs (95% confidence intervals) and p-values were obtained via a logistic regression analysis.

OR, Odds ratio. CI, Confidence interval.

CPAOA, Cardiopulmonary arrest on arrival. ICU, Intensive care unit. ECMO, Extracorporeal membrane oxygenation.

**Supplementary Table 4.** Multivariable analysis of factors associated with mortality in hospitalized patients

**Model 1**

| Variables | Adjusted OR (95% CI) | *p* value |
| --- | --- | --- |
| Male | 1.19 (0.85 - 1.68) | 0.31 |
| Infant (< 1 year) | 1.3 (0.91 - 1.88) | 0.15 |
| CPAOA | 25 (16.5 - 37.8) | **<0.01** |
| Low-volume hospital | 1.73 (1.10 - 2.70) | **0.02** |

**Model 2**

| Variables | Adjusted OR (95% CI) | *p* value |
| --- | --- | --- |
| Male | 1.28 (0.90 - 1.83) | 0.18 |
| Infant (< 1 year) | 1.11 (0.76 - 1.63) | 0.57 |
| CPAOA | 15.2 (9.79 - 23.7) | **<0.01** |
| Low-volume hospital | 1.6 (1.01 - 2.54) | **0.04** |
| Mechanical ventilation | 10.5 (6.11 - 17.9) | **<0.01** |
| Renal replacement therapy | 3 (1.74 - 5.17) | **<0.01** |

ORs (95% confidence intervals) and p-values were obtained via a logistic regression analysis.

Adjusted values were obtained from a logistic regression analysis after adjusting for all variables listed in each model.

OR, Odds ratio. CI, Confidence interval.

CPAOA, Cardiopulmonary arrest on arrival.
